# Adaptability of Napiergrass (*Pennisetum purpureum* Schumach.) for Weed Control in Site of Animals Buried after Foot-and-Mouth Disease Infection

**DOI:** 10.1155/2016/6532160

**Published:** 2016-05-04

**Authors:** Yasuyuki Ishii, Yusuke Iki, Kouhei Inoue, Shuhei Nagata, Sachiko Idota, Masato Yokota, Aya Nishiwaki

**Affiliations:** ^1^Faculty of Agriculture, University of Miyazaki, Miyazaki 889-2192, Japan; ^2^Division of Livestock Production Research and Support, Center for Animal Disease Control, University of Miyazaki, Miyazaki 889-2192, Japan; ^3^Takanabe Agricultural High School, Miyazaki 884-0006, Japan

## Abstract

After the infection of foot-and-mouth disease outbreaks in Miyazaki, Japan, in 2010, cattle and swine were slaughtered and buried in a site of 100 ha, where weed control is difficult and costly since lands are unlevelled and prohibited to be plowed for 3 years. To consider the adaptability of napiergrass (*Pennisetum purpureum* Schumach.) to the animal burial site for weed control, two napiergrass varieties, normal Wruk wona (WK) and dwarf late-heading variety (DL), were transplanted, compared with sowing of maize (MZ) and sorghum (SR) in both burial (BU) and neighboring bordered area (BO) in mid-June 2011. Even though several weed control methods were subjected to lands, MZ and SR failed to be established stably at only 1/3–1/2 due to the suppression of growth by indigenous weeds, while WK and DL successfully established as high as 82–91% and 73–85%, respectively, in 2011. The poor establishment of MZ and SR after sowing tended to be increased with the year from establishment. Plant dry matter yield and cellulose concentration were the highest in WK in 2011, while overwintering ability was constantly higher in DL in the 3 years. It is necessary to consider the utilization of forage plants on the animal burial site.

## 1. Introduction

After the infection of foot-and-mouth disease (FMD) outbreaks in Miyazaki Prefecture in 2010, around 68 thousand head of cattle and 220 thousand swine were slaughtered and buried in 252 sites, reaching up to 100 ha (around 97.5 ha) [1]. Since the FMD is a highly contagious disease among bovine diseases [2], the Japanese national law ordered to slaughter and bury cattle and swine in the infectious regions and prohibited plowing the sites where slaughtered animals were buried for 3 years [3]. The burial sites are difficult and costly for weed control, since lands are unlevelled and plowing for 3 years is prohibited [3]. Miyazaki Prefectural Takanabe Agricultural High school is located in Koyu District where FMD broke out severely. In the school, 32 dairy cows, 22 beef cows, and 281 swine were buried after slaughtering, and thus weed control on the burial sites was difficult to be conducted. Another concern is the soil pollution caused by the large amount of minerals being effluent from the decomposition of buried animals by soil microorganisms [3].

Napiergrass, especially dwarf variety of late-heading type (DL), has high adaptability for grazing use by dairy and beef cows [4] and has superior regional adaptability to several prefectures in southern Kyushu [5]. Since the grass, moreover, had superior absorbing capacity in several inorganic elements [6–8] and was fit to the remediation of soil environments [9], it is considered to be a suitable forage crop for weed control in the animal burial site where large amount of minerals is concerned to be eluted to the neighboring environments [3] and machinery operation is hard due to the elevated and unlevelled land surface. Normal type of napiergrass has a perennial habit with high dry matter production potential in low-altitudinal sites of southern Kyushu [10] and dwarf DL is superior in utilizing as a roughage [4], is easy for controlling sward management [3], and is suitable for the sloped land of southern Kyushu [5]. Weed damage on yield loss was severe especially at the establishment in both maize [11, 12] and napiergrass [13].

Therefore, in the current study, normal and dwarf varieties of napiergrass were established only once in May-June 2011 by inserting nursery plants into land surface by hands and maize and sorghum by sowing in every spring during the period from 2011 to 2013, so as to consider the adaptability of napiergrass (*Pennisetum purpureum* Schumach.) to the animal burial site for weed control in the region.

## 2. Materials and Methods

### 2.1. Experimental Site and Cultivation of the Species

The experiment was conducted in the animal burial (BU) site sized at 9-10 m width × 63-64 m length (around 620 m^2^), where 32 dairy cows, 22 beef cows, and 281 swine were buried after slaughtering, and the neighboring border (BO) area (around 1180 m^2^), which was managed together with BO site before the FMD outbreaks, is located in Maizuru Ranch, Takanabe Agricultural High School (31.5°N, 131.5°E, 71 m a.s.l.). The consecutive three-year experiment was carried out during the period from June 2011 to February 2014. The previous vegetation of native annual weeds such as* Digitaria* spp. was treated with cutting by hand-mowing machine, followed by glyphosate herbicide (Monsanto Co. Ltd.) at 4.4 mL·m^−2^ on June 6, 2011. The BU sites were divided into 3 plots, where the sanitizing and slaughtering materials were mainly buried in plot 1 and the animals were mainly buried in plot 3, and the intermediated areas were named as plot 2, and 1 m wide space was set between plots. The BO areas were directly connected to BU areas and four grass species such as two napiergrass varieties, normal type of Wruk wona (WK) and dwarf variety of late-heading type (DL), maize (cv. Ohka, MZ), and sorghum (cv. Sanjaku sorgo, SR) were plotted with randomized block design with three replications. Each grass species had four rows in each replication, and, thus, each plot for each grass species had 4 m width × 9-10 m length for BU area and 4 m width × 53-54 m length for BO area.

On June 14, 2011, two types of napiergrass, WK and DL, were established by hand-transplanting at 1 plant m^−2^ (1 m interrow and 1 m intrarow spacing) in contrast with hand-sowing of MZ and SR at 5 plants m^−2^ (1 m interrow and 0.2 m intrarow spacing) both in the BU and BO areas at Takanabe Agricultural High School. Soil chemical property, establishment and sustainability of plants, plant growth, and structural carbohydrate composition of plants were determined only for the BU areas in 2011 and for both BU and BO areas in the growing season of 2012 and 2013. On June 14, 2011, only for BU areas, peat-moss (pH 4.0) was supplied at planting spot with the rate of 650 mL·m^−2^ in order to decrease soil pH due to the heavily accumulated slacked lime for the regulation of FMD disease. Chemical compound fertilizer was supplied in early July every three years at 4.5 g·m^−2^ of each of N, P_2_O_5_, and K_2_O on July 1, 2011, July 3, 2012, and July 10, 2013. After wintering on March 14, 2012, February 23, 2013, and February 24, 2014, forages, composed of dead leaves and stems suffered from frosts, were cut by hand-mowing machine and transported outside of the BU and BO areas and then labor-power for this practice was monitored only on March 14, 2012. After wintering on June 16, 2012, and June 26, 2013, two napiergrass species were retransplanted at the spot of no-regrowth and seeds of MZ and SR were sown again, and intrarow spaces were cut with hand-mowing machine on July 2, 2012, July 3, 2012, and July 10, 2013, while no other sward management was supplied thereafter in each year.

### 2.2. Soil and Plant Observation

At the beginning of this trial on June 14, 2011, and at the end of the following two years on November 7, 2012, and November 16, 2013, top layer soils below 10 cm from the ground surface were sampled at 3–6 sites mixed for chemical soil properties in each grass species and plot in both BU and BO areas. Soil chemical properties, such as pH and electric conductivity (EC), were measured by pH meter (Model PRN-41, Fujihira Co. Ltd., Tokyo) and by EC meter (Model B-173, Horiba Co. Ltd., Kyoto), respectively.

At about two weeks after the transplanting of WK and DL and the sowing of MZ and SR in each year on July 1, 2011, July 3, 2012, and July 10, 2013, the early plant establishment was determined and at the end of each growing season on December 20, 2011, October 27, 2012, and November 15, 2013, the final plant establishment was determined in both BU and BO areas. Plant overwintering was determined on June 9, 2012, and May 27, 2013. Plant growth properties such as plant height and tiller number were determined from four plants and fresh weight from one plant in each species and replication only for the burial (BU) areas in 2011 and for both BU and the (neighboring) bordered (BO) areas in the growing season of 2012 and 2013. Subsample was taken from around 1 kg of fresh weight in every species and replication and dry weight was calculated by the percentage of dry weight of the subsample. Dry matter yield was determined by plant dry weight multiplied by plant density and percentage of the final plant establishment.

### 2.3. Structural Carbohydrate and Digestibility

Dry sample of each grass species was ground through 1 mm screen and determined for plant structural carbohydrate such as neutral detergent fiber (NDF), acid detergent fiber (ADF), and acid detergent lignin (ADL) by fiber analyzer (Model A200; ANKOM Technology Corp., Macedon, NY, USA). Cellulose and hemicellulose content were calculated by the difference between NDF and ADF and between ADF and ADL, respectively [14].* In vitro* dry matter digestibility (IVDMD) was determined by pepsin-cellulase digestion assay [15] using* in vitro* incubator (Model D200; ANKOM Technology Corp., Macedon, NY, USA).

### 2.4. Statistical Analysis

One-way analysis of variance was carried out using SPSS for Windows ver. 16.0 software (Chicago, IL, USA). Differences in means among plots were evaluated using the least significant difference test at the 5% level.

## 3. Results and Discussion

### 3.1. Soil Chemical Property

**Figure 1:**
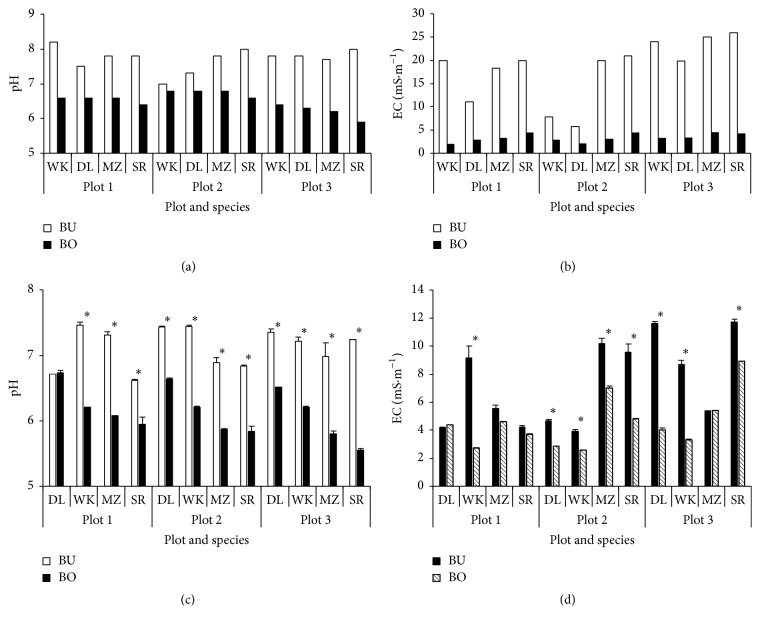
Changes in soil pH (a) and soil electric conductivity (EC, (b)) at the harvest on November 7, 2012, and soil pH (c) and soil EC (d) on November 16, 2013. For plots 1–3, animals were mainly buried in plot 3, sanitizing and slaughtering materials were mainly buried in plot 1, and both of animals and materials were buried in plot 2. Abbreviations of species are as follows: napiergrass cv. Wruk wona (WK), and dwarf variety of late-heading type (DL), maize (cv. Ohka, MZ), and sorghum (cv. Sanjaku sorgo, SR). *∗* shows the difference between BU and BO areas at 5% level.

The pH and EC in the top soil are shown in Figures 1(a) and 1(b), respectively, for both BU and BO areas. The pH values were apparently higher in BU areas ranging from 7 to 8 than in BO areas ranging from 6 to 7. The Japanese law states that the slaughtered animal should be buried at more than 1 m depth and the slaked lime was used on the surface of the pits and on top of the carcasses beneath the soil cap [3], affecting higher pH in BU than in BO areas. However, the difference in pH among replications was not apparent and that between BU and BO areas tended to be reduced with years (Figure 1(a)). The difference in EC values between BU and BO areas tended to be the same. Having higher values for EC in BU than in BO areas below 5 mS·m^−1^ was almost comparable with the measurement under digested effluent application [8]. The difference in EC between BU and BO areas tended to increase from plot 1 to plot 3, reflected by the difference in buried animal concentration (Figure 1(b)).

### 3.2. Plant Overwintering

**Figure 2:**
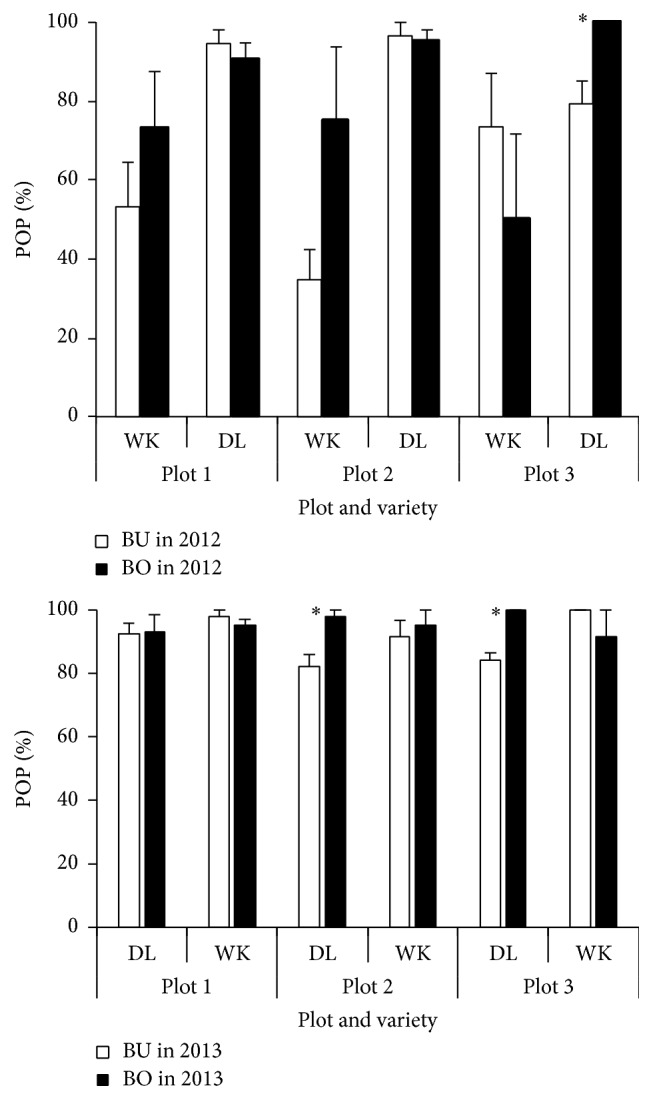
Percentage of overwintered plants (POP) of two napiergrass varieties on June 9, 2012, and on May 27, 2013. For both plots and varieties, refer to Figure 1. *∗* shows the difference between BU and BO areas at 5% level.

Overwintering of overwintered plants (POP) in spring 2012 is shown for both BU and BO areas in Figure 2. POP in DL had higher values than other grasses, especially showing more than 90% in both plot 1 and plot 2, while POP in WK was lower than DL, especially showing 35% in BU area at plot 2. No differences in POP were detected between BU and BO areas, except for the difference in DL at plot 3 in 2012. The lower POP in WK was consistent with the previous research in the region [10], compared with DL [16].

### 3.3. Plant Establishment

**Figure 3:**
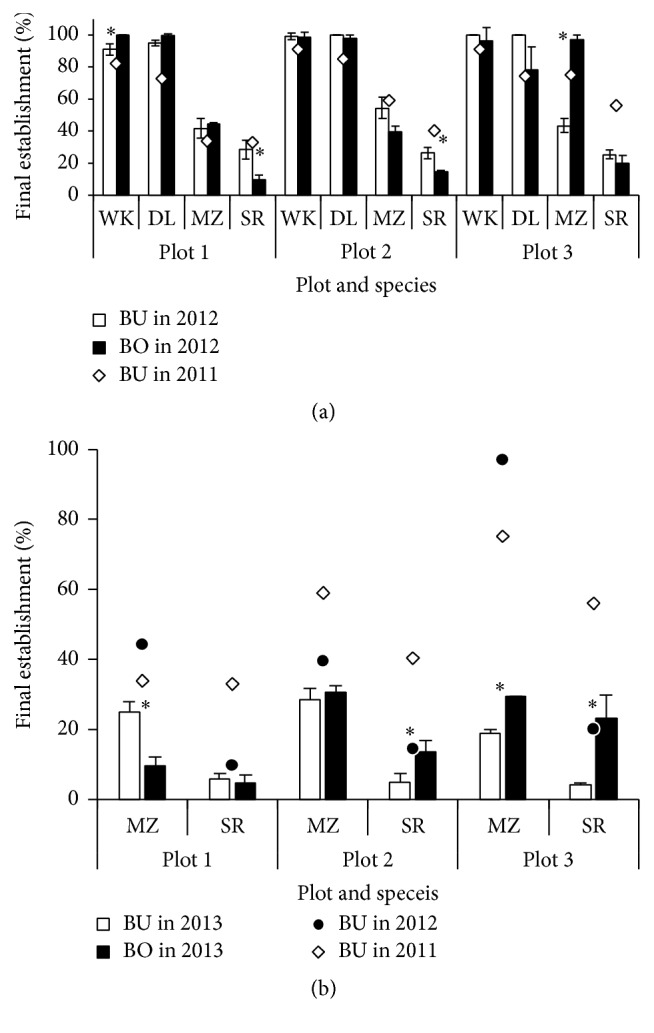
Final established percentage of 4 species in late autumn on December 20, 2011, and October 27, 2012 (a), and of 2 annual species on December 12, 2011, October 27, 2012, and November 15, 2013 (b) (mean ± standard deviation). *∗* shows the difference between BU and BO areas at 5% level. For both plots and species, refer to Figure 1.

Percentage of plant establishment in late autumn of each year is shown in Figure 3 for BU area in 2011 and for each of BU and BO area in 2012 and 2013. Early plant establishment in 2011 was higher than 95% in two napiergrass varieties, DL and WK, followed by the significant decrease to 87–90% in MZ and 36–74% in SR. Percentage of the final plant establishment on December 20, 2011, was the highest at 90% in WK, followed by 80% in DL, which was consistently high with that under no weed control in the DL pasture [13]. However, the percentage of annual crops decreased significantly to 34–75% in MZ and 33–56% in SR, showing the great variability in the latter species among plots. The two annual crops, MZ and SR, had the highest percentage in plot 3, followed by plot 2 and plot 1, reflected by the suppression of annual weeds such as* Digitaria ciliaris* in plot 3. The tendency continued in 2012 and 2013, suggesting the perennial and labor-saving sward control in both BU and BO areas against weed invasion only by hand-planting of the two napiergrass varieties at the establishment.

### 3.4. Growth Attributes

**Figure 4:**
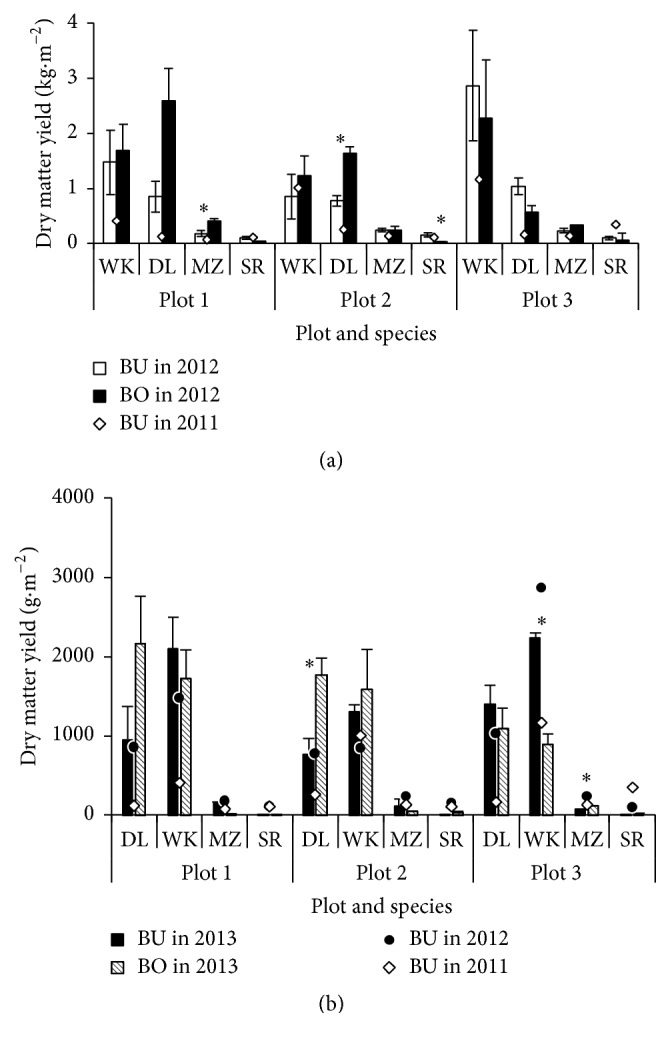
Dry matter yield at the harvest in late autumn in BU area on December 20, 2011, and in both BU and BO areas on November 3, 2012 (a), and in both BU and BO areas on November 15, 2013 (b) (mean ± standard deviation). *∗* shows the difference between BU and BO areas at 5% level. For both plots and species, refer to Figure 1.

Plant dry matter yield at the harvest is shown in Figure 4 for BU area in 2011 and for each of BU and BO areas in 2012 and 2013. Plant dry matter yield was the highest in plot 3, followed by plot 2 and plot 1 among the examined grass species, except for DL at plot 3, suggested for buried animal concentration in plot 3. However, the carcasses were buried below more than 1 m from the soil surface by the regulation of the Japanese law [3], leading to a large limitation of napiergrass in extracting minerals from decomposition of the carcasses, since root penetration of napiergrass was not significant in the zone where soil microorganisms were breaking down the decomposition products of slaughtered animals. Plant dry matter yield was the highest in WK, reflected by high plant height and leaf area index [17], while the difference among the other three grass species was small in 2011. In 2012, the difference in plant dry matter yield among species tended to enlarge, showing high yield in WK followed by DL, while the yield was severely limited in the two annual species. Plant dry matter yield in BU area tended to be lower than that in BO area in plots 1 and 2 due to alkaline soils, while the tendency in dry matter yield between BU and BO areas turned to be reverse in plot 3, possibly due to the accumulation of nutrients from buried animals, except for no difference in MZ. The yield potential in BU area was reduced especially in the established year from the previous research in the region [16], while it increased to the comparable levels in the following year.

### 3.5. Structural Carbohydrates and Digestibility

**Figure 5:**
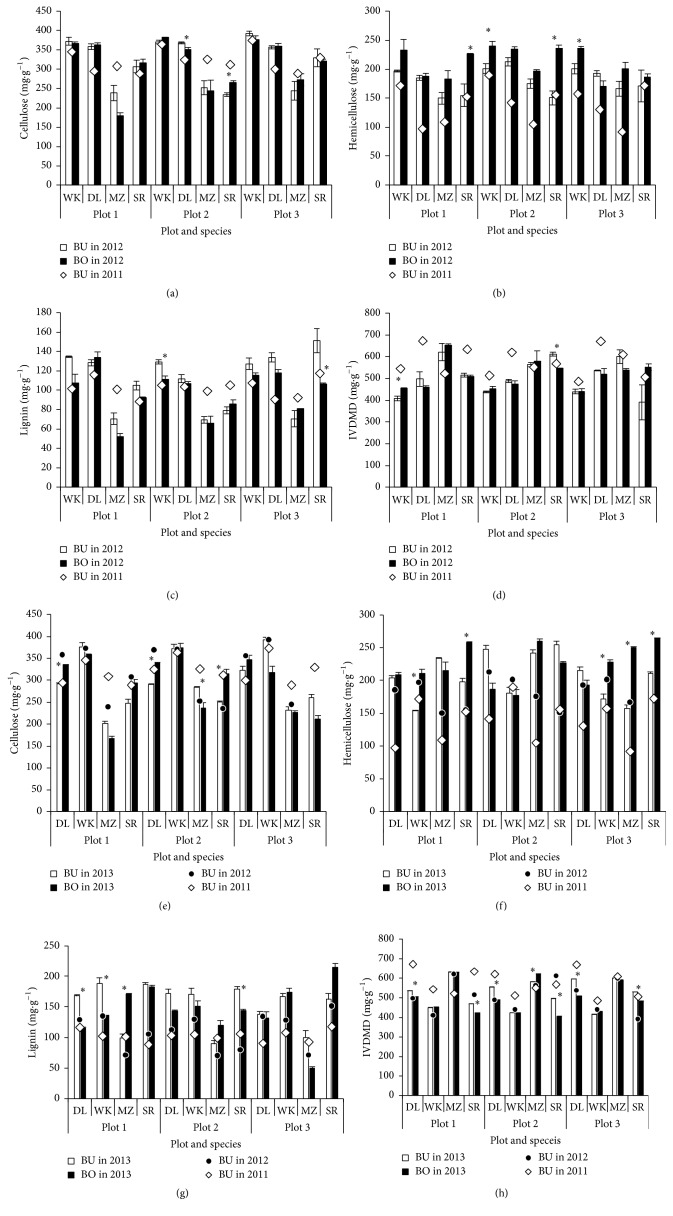
Cellulose (a), hemicellulose (b), and lignin (c) contents and* in vitro* dry matter digestibility (IVDMD (d)) of 4 species in late autumn on December 20, 2011, and on October 27, 2012 (mean ± standard deviation). *∗* shows the difference between BU and BO areas at 5% level. For both plots and species, refer to Figure 1. Cellulose (e), hemicellulose (f), and lignin (g) contents and* in vitro* dry matter digestibility (IVDMD (h)) of 4 species in late autumn on December 20, 2011, November 27, 2012, and November 15, 2013 (mean ± standard deviation). *∗* shows the difference between BU and BO areas at 5% level. For both plots and species, refer to Figure 1.

Contents of structural carbohydrates in cellulose (Figures 5(a) and 5(e)), hemicellulose (Figures 5(b) and 5(f)), and lignin (Figures 5(c) and 5(g)) are shown for only BU area in 2011 and for each of BU and BO area in 2012 and 2013. Cellulose content was the highest in WK, reflected by highest dry matter yield, while it was not different among the other 3 species, having the positive correlation between dry matter yield and cellulose content (*r* = 0.892, *P* < 0.01) in all species and plots. Cellulose content in WK was the highest in plot 3, followed by plot 2 and plot 1. Hemicellulose content was higher in WK and SR than DL, showing the lowest content in MZ (Figure 5(b)). Lignin content did not differ so much among species, ranging from 8.8 to 11.7% as in 4–8-month cutting [14], and it tended to increase with the increase in dry matter yield for SR, while no apparent tendency between dry weight and lignin content was detected in the other species (Figures 5(c) and 5(g)). Compared with the research conducted in Thailand for determining the cutting interval ranging from 1 to 12 months, normal napiergrass variety of common and dwarf variety Muaklek showed cellulose content at 42.3 and 39.1%, respectively, and hemicellulose content at 24.4 and 22.8%, respectively [18], which was higher in structural carbohydrate than the current data for normal Wruk wona and dwarf DL varieties. Content of structural carbohydrate such as cellulose tended to increase with growth [18], while these ontogenetic changes tended to retard in BU area.* In vitro* dry matter digestibility (IVDMD) was shown in Figure 5(d), being the highest in DL at 65.4 ± 2.9% (mean ± standard deviation, *n* = 3), followed by SR (56.9 ± 6.4%), MZ (56.1 ± 4.4%) and the lowest in WK at 51.4 ± 2.9%, which was variable with cutting interval [14, 19].

### 3.6. Weed Control

Napiergrass variety WK almost occupied the BU area perfectly in mid-November, when MZ was suppressed by weeds under no weed control [11]. In mid-March after wintering in 2012, leafage in all grass species was dead by the frost to fall down by the winter seasonal wind. However, two napiergrass species (WK and DL) covered the ground by dead leaves, which completely suppressed the growth of spring weed. It took 74 man hour ha^−1^ to do the harvest of whole BU and BO areas (1,800 m^2^) and to transport dead leaves on the ground to the outside of the area, monitored on March 14, 2012.

### 3.7. Future Subjects

Napiergrass can be overwintered in the low-altitudinal areas, including the present experimental site [5], while the growth of two napiergrass varieties tended to be retarded in BU area, compared with BO area. Japanese Act on Domestic Animal Infectious Disease Control prohibited plowing the land of animal burial site for three years up to mid-May 2013 and thus the data in the present research work should be preferred to be utilized efficiently as one example of weed control management on BU site. In the present study, the suitability and adaptability of napiergrass cultivation on BU area were almost verified in low-altitudinal sites of Kyushu. In a near future, it will be necessary to consider the safety of cultivated crops on BU area to animal feeding by the risk assessment of disease transmission, public perception, and cost management of napiergrass cover on the burial sites.

## 4. Conclusions

Two napiergrass varieties of DL and WK in the present research can be overwintered at more than 90% on the animal burial site, which was similar to the case in the low-altitudinal areas of southern Kyushu. Suppression of napiergrass growth on the BU area, compared with the BO area, was weakened with time and the two napiergrass varieties successfully suppressed the growth of weedy species, while annual forage crops of MZ and SR failed to do the suppression of weed. Soil chemical properties of pH and EC on the BU area tended to decrease with years to be a weak alkaline condition with less than 30 mS·m^−1^ of EC, which was lower than the soil remediation level, suggesting that mineral accumulation to the top soil by the burial of animals was limited in three years of the experiment. Future use for herbage production on the burial sites would be solved by establishing public consensus based on the scientific and economic risk assessment.
